# Investigating the effect of sudden occlusion of the testicular vessels on the testicular tissue in rat models

**DOI:** 10.1097/MS9.0000000000000976

**Published:** 2023-06-17

**Authors:** Reza Shojaeian, Mehran Hiradfar, Pegah Bahrami Taqanaki, Sarina Khorsand Ahmadi, Yousef Jelveh Masouleh, Leila Ameri, Mahdi Parvizi Mashhadi

**Affiliations:** a Department of Pediatric Surgery; b Mashhad University of Medical Sciences; c Parsian imaging center, Mashhad; d Akbar Children’s Hospital; e University of Medical Sciences, Azadi Square, Mashhad, Khorasan Razavi, Iran

**Keywords:** cryptorchidism, Fowler–Stephens, histopathology, necrosis, orchiopexy

## Abstract

One of the ways to treat undescended testicles is to use orchiopexy surgery, which is modified into the two-stage Fowler–Stephens technique in cases of short or immobile testes. The disadvantage of using this technique is the probability of testicular ischaemia following the sudden closure of the testicular artery; although the collateral arteries prevent atrophy, the testis loses its germ cells during this stress and may no longer be functional. Therefore, this study aims to examine the changes in testicular tissue regarding necrosis and infarction after the occlusion of the vessels.

**Methods and materials:**

In this experimental study 15 male rats weighing 200–250 g were prepared and first, one of the rats was sacrificed and testicles on both sides were used for pathology control. After general anaesthesia vascular ligature was performed with the left testes undergoing both venous and arterial occlusion and the right testes only arterial occlusion. After 1 month, all specimens were killed and the testes were completely removed and sent for histopathological evaluation.

**Results:**

A total of 14 rats and 28 testes were studied in two equal groups of case and control. There was no significant difference between the case and control groups in terms of testicular volume, consistency, and viability. Microscopic findings revealed that necrosis, infarction, and state of inflammation were significantly higher in the case group than in the control group.

**Conclusion:**

The results of this study show that abrupt closure of the testicular artery in rats is associated with necrosis and infarction, decreased spermatogenesis, and more inflammation. However, no significant differences were found in terms of macroscopic findings including volume, consistency, and viability.

## Introduction

Highlights

- Abdominal undescended testis with short vessels remains an issue for paediatric surgeons worldwide. The two-staged Fowler–Stephens technique that is used for this type of cryptorchidism puts the testes at risk of infarction due to the shortage of blood supply. Many studies have been conducted on the size, viability, and consistency of the testes after the two-staged Fowler–Stephens surgical technique for short or immobile undescended testes. The results of these studies are summarized in the manuscript.
- This study in addition to examining the volume, consistency, and viability of the testes, also deals with the less discussed topic of histopathological changes in testicular tissue after vascular occlusion.
- The results of our study show that abrupt closure of both testicular artery and vein in rats is associated with necrosis and infarction, decreased spermatogenesis and more inflammation in histopathological evaluations.

Cryptorchidism is a term used to describe a condition where one or both testes are absent in the scrotum. This absence is more common on the right side^1^. Cryptorchidism has various classifications including; unilateral or bilateral, palpable or non-palpable, and congenital or acquired^1,2^. When the undescended testicle is anywhere in the inguinoscrotal area, it is called a palpable cryptorchidism. An ectopic testis, testicular agenesis, and vanishing testis include the causes of non-palpable testicles^3^.

One of the ways to treat an undescended testicle is using orchiopexy surgery. This technique is used for a palpable undescended testicle or an ectopic testicle. One-stage orchiopexy is used with testes of normal size and with sufficient length of testicular blood vessels^4,5^. Various surgical techniques have been introduced to treat non-palpable undescended testes including the one-stage or two-stage Fowler–Stephens, autotransplantation, and even the standard orchiopexy technique for non-palpable but close to the internal ring testes^6^.

Fowle–Stephens technique was first introduced in 1959 by Fowler and Stephens as a treatment option for high intra-abdominal testes with short vessels^7^. In this technique, first, the testicular artery and internal spermatic vein are ligated and after 6 months and once the collateral blood supply of the testis is provided from the vasal vessels, the testicle is separated and placed inside the scrotum. The problem with this technique is testicular ischaemia following the sudden closure of the testicular artery, although collateral vessels prevent testicular atrophy, the testis loses its germ cells during this stress and may no longer be functional^8^.

According to previous reports, the success of this procedure was assessed by the return of the testis to the scrotum, changes in testicular volume, and testicular viability^9,10^. In addition, a study suggests that the fertility rate of corrected bilateral intra-abdominal testes by the Fowler–Stephens technique is less than that of the one-sided undescended testis^11^. Several experimental animal studies on rats have been performed over the years, mostly evaluating the volume and viability of the testes after the procedure. However, in this study changes in tissue and germ cells, testis size, consistency, and viability are assessed in the testicles of the same rats with the right testis having ligation on only the right testicular artery and the left on both testicular artery and vein.

As a more detailed examination of the advantages and disadvantages of this technique is required, this study aims to provide a model of this technique and its effect on the testicular tissue and the histopathologic changes following the surgery in rat models.

## Materials and methods

This is an experimental study on basic science conducted on adult male rats between March 2018 and March 2022. This study was approved by the Ethics Committee with number 971169.

### Study design

Experimental study on adult male rats. A total of 15 male rats were chosen. One rat was initially sacrificed to be used as a normal histopathologic sample, then 14 testes were allocated in each groups of case and control with the left testes in the case group and right testes in the control group. Case and control groups were randomly chosen.

Case group: All the left testes of all 14 rats which were planned to undergo ligature of both testicular vein and artery.

Control group: All the right testes of all 14 rats which were planned to undergo ligature of only the testicular artery.

### Materials

Fifteen adult male rats with an average weight of about 200–250 g. Rats were kept in a conventional cage with an 800 cm2 floor area and a height of 18 cm. The cage included a solid floor and a pathogen-free environment. Rats were kept at a temperature of 22 ± 1 °C with a cycle of 12 h of light and 12 h of darkness with free access to food and water. Cage cleaning was carried out in the daytime. To increase welfare playpens were used. To enrich their environment, shelters with one entrance were installed for them, and the necessary care was taken to maintain the communication between the rats as well as with humans, which included not isolating the rats, playing with them, and tickling them.

### Study aim

Investigating histopathological changes in the rat testis following sudden occlusion of the testicular artery.

### Intervention

Testicular artery occlusion in rats.

### Variables

The variables of this study included the two groups of quantitative and qualitative variables. The quantitative variable of the study was testicular size. Testicular size is reported in millimetres and measured by a caliper. Qualitative variables were, the state of testicular consistency, viability of testes, the state of seminiferous tubules, the state of spermatogonium, and the degree of inflammation.

### Implementation methods

Fifteen male rats with an average weight of 200–250 g were prepared. The sample size was arrived at by studying of similar studies done on this topic. Ketamine plus Xylazine was injected intraperitoneally at a dose of 60 and 5 mg/kg, respectively, for anaesthesia. While, placed in the supine position, one of the rats was sacrificed, and both testes were used as a histopathologic control sample. All the remaining rats were also placed in the supine position and their abdominal and inguinal areas were shaved and disinfected with betadine. Then depletion and cleansing of their abdominal, inguinal and scrotal areas was carried out. Consequently, an incision was made at the lower abdominal region of all rats to expose the muscles and vessels of the area.

Then, ligature of the left testicular artery and vein of the left testis in all rats was done. On the right side only ligature of the right testicular artery was done. Ligation of the vessels was performed using 4-0 silk sutures. After repairing the area of skin where incisions had been made with 5-0 sutures, animals were followed for one month in a suitable environment. After one month, all the samples were sacrificed and the testicles were removed and placed in Bouin’s solution and sent to the pathology department for histopathological evaluation. The pathologist examining the tissues did not have any knowledge on the tissue belonging to the case or control group. The presence of germ cells and tissue changes were factors that were evaluated in the histopathologic assessment. Finally, comparisons regarding the testis size, testis consistency, and testicular viability in case and control groups were done.

### Statistical methods and sample size

Data were entered into Prism software and analyzed. Independent *t*-test and Fischer’s exact test were used to analyze the data. In all the statistical tests used, *P* value less than 0.05 was considered as a significant level.

### Ethical considerations

All experiments and procedures performed on rats were in accordance with the ARRIVE guidelines^12^. Sacrificing rats was done in a closed chamber and by a cotton soaked in ether, so that the animals would not suffer pain.

## Results

A total of 14 rats with 28 testes were studied in two equal groups of case and control. In all steps of analysis 14 animals were evaluated with 14 testes in case group and 14 testes in control group.

No adverse events occurred during any of the procedures abolishing the need to modify the experimental protocols planned ahead.

At first, testicular volume was evaluated in both control and case groups at each operation session. There was no significant difference between the case and control groups in terms of testis volume in the first (*P*=0.417) and second operation (*P*=0.137) (Table 1) (*t*-test was used for comparison).

**TABLE 1:** Comparison of testicular volume in case and control groups.

	Control group standard±mean deviation	Case group standard±mean deviation	*p*
Testis volume in 1^st^ operation	2.67±1.21	1.76±2.56	0.417
Testis volume in the 2^nd^ operation	2.64±1.17	2.37±1.93	0.137

**Figure 1:**
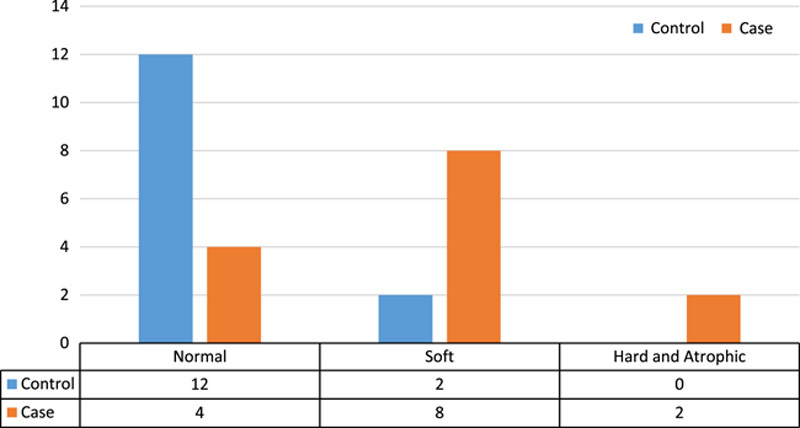
Comparison of testicular consistency in case and control groups.

Next, the consistency of the testes was evaluated in both control and case groups. As can be seen in Figure 1, there is no significant difference between the two groups in terms of testicular consistency (*P*=0.109) (Fisher’s exact test was used for comparison).

**Figure 2:**
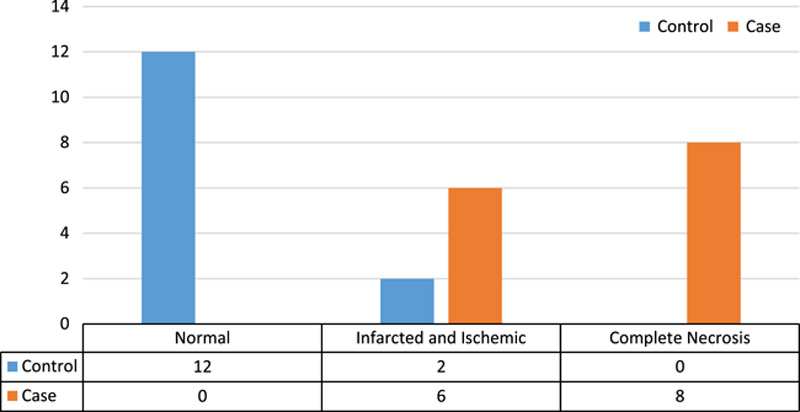
Comparison of microscopic findings of testicular seminiferous tubules in case and control groups.

The results of microscopic examination of seminiferous tubules in both control and case groups showed that necrosis and infarction was more significance in the case group than in the control group (*P*<0.05). The results of this comparison can be seen in Figure 2 (Fisher’s exact test was used for comparison).

**Figure 3:**
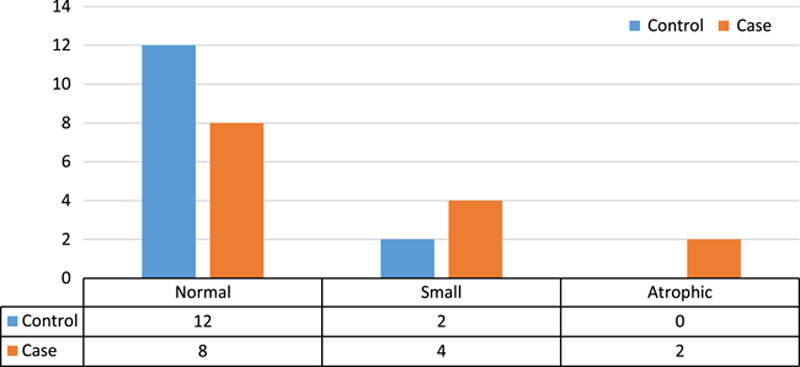
Comparison of testicular viability in case and control groups.

The results of examining the viability of testes in both control and case groups showed that there was no significant difference between the two groups (*P*=0.249). The results of this comparison can be seen in Figure 3 (Fisher’s exact test was used for comparison).

**Figure 4:**
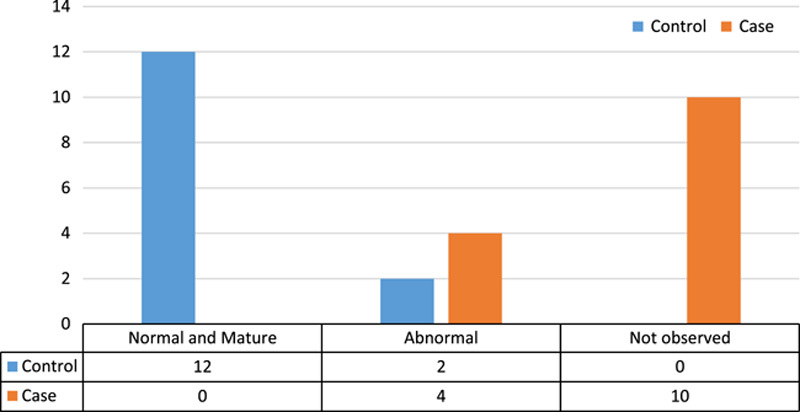
Comparison of state of spermatogonia in case and control groups.

Assessment of the state of spermatogonia in both control and case groups showed a significant difference (*P*<0.05). The results of this comparison can be seen in Figure 4 (Fisher’s exact test was used for comparison).

**Figure 5:**
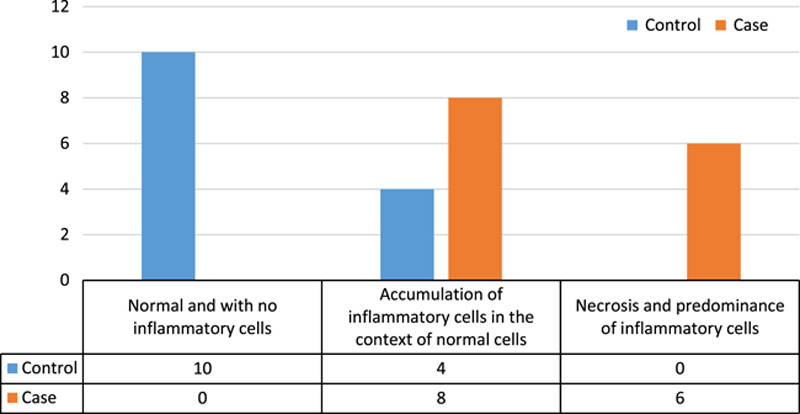
Comparison of the degree of inflammation in case and control groups.

Evaluation of the degree of inflammation in both control and case groups showed that the degree of inflammation in both case and control groups had a significant difference (*P*<0.05). The results of this comparison can be seen in Figure 5 (Fisher’s exact test was used for comparison).

## Discussion

Cryptorchidism is a common genitourinary condition in infants ranging between 1 and 3% of full-term male babies and around 30% of pre-term babies^11^. Due to the physiologic surge of gonadotropins in the first 2 months, a natural descent in the testes might occur causing a decrease in the disease’s incidence in the first year of age^13^.

Complications of an undescended testicle include an inguinal hernia, testicular torsion, testicular trauma, infertility, and testicular cancer^14^. Since Fowler and Stephens first introduced the Fowler–Stephens technique, many modifications have been made to this technique to make it more efficient. It was originally described as a method using division of spertmatic vessels during orchiopexy to treat intra-abdominal testes, using the assistance of collateral blood vessels. Later Ransley and colleagues proposed a modified two-staged Fowler–Stephens technique to increase the opportunity of collateral vessels formation^7^. To this day many studies have been conducted on adverse effects of this technique, mainly focusing on the testicular volume, consistency, mobility, and fertility state of the testis^9^. In the current study we have assessed histopathologic changes in the testes undergoing vascular occlusion alongside the mentioned measures in the other studies.

In this study total of 14 rats and 28 testes were examined in two equal groups of case and control. Testis volume in two groups was evaluated and there was no significant difference between the two groups in terms of testis volume in both operation sessions. Also, there was no significant difference between the case and control groups in terms of testicle consistency. The results also showed no significant difference between the two groups regarding testicular viability in both control and case groups and the state of spermatogony in both control and case groups were also similar.

In the study of Pascual *et al.* ^10^, which aimed to investigate the effect of closing the testicular artery on changes in the collateral vessels and also the testis viability of rat testis was done, the results showed no significant difference between the average testicular volume and viability before and after surgery, which is in concordance with our study. Rosito *et al.* ^9^ also did not observe any changes in testicular volume in their study of 44 intra-abdominal testes undergoing stage 1 and 2 of Fowler–Stephens technique. In another study performed on 15 patients with intra-abdominal undescended testis, the postoperative results showed that only one patient experimented testis volume loss after the second stage^15^. There have been, however, studies indicating volume loss after the second stage of the surgery. A study on 76 non-palpable testes with 36 testes with volume assessment revealed that previously normal sized testes (after the first stage) showed volume loss after the second stage^16^. Esposito *et al.* ^7^ followed 12 patients with high intra-abdominal testes for 10–17 years after the surgery revealing volume reduction in all patients compared to the same patients’ normal descended testis. The results of this study is not in concordance with our findings.

In another study conducted by Pascual *et al.* ^17^ with the aim of investigating the effect of testicular ligation before puberty on testicular function, the results revealed that a decrease in testicular blood flow during the pre-puberty period of the rat does not change the weight and function of the testicle). These findings can indicate the importance of performing the two-step Fowler–Stephens technique at a younger age.

Lee and colleagues, showed that use of human chorionic gonadotropin before the Fowler–Stephens surgery was in bilateral cryptorchid rats causes an average increase in testicular volume^18^.

Our study showed a significant reduction in the number of spermatogonia in the case group, which is in accordance with studies performed by Rosito and colleagues and AbouZeid and colleagues^9,19^. In the paper by Rosito and colleagues it was demonstrated that for the subgroup of younger patients who had higher numbers of spermatogonia per tubule at stage 1, the spermatogonia were significantly lower at stage 2 surgery compared to stage 1 and AbouZeid and colleagues showed that in 20 intra-abdominal human testes, there was a decrease in spermatogonia per tubule numbers at stage 2. However, another study has depicted no significant difference between the spermatogonia per tubule number in the two stages in 13 intra-abdominal human testes^20^.

It should be noted that in our study, the state of spermatogonia is reported as absent (decrease in number) and immaturity, and not reporting number of spermatogonia per tubule number is one of the limitations of this study.

The current study also analyzed the histopathologic changes of the testes before and after the vascular occlusion, which is a topic that has been less discussed. Microscopic findings of our study showed significantly more necrosis, infarction, and inflammation in the case group than in the control group (*P*<0.05).

Tanley and colleagues, discussed the effect of closing the testicular arteries on the nitric oxide levels, inducible nitric oxide synthesis, endothelial nitric oxide synthesis, and specific apoptosis of germ cells; The results showed that 24 h after performing the Fowler–Stephens orchiopexy technique, the level of nitric oxide in both testes of the intervention group was increased compared to the control group. Expression of endothelial nitric oxide synthesis was clearly increased in the operated testis, while there was a slight increase in the contralateral testis at 24 h after surgery. Increased apoptosis was also clearly seen in the operated testis, while it was seen only in a limited number of cells on the opposite side 24 h after surgery. This study states that using Fowler–Stephens orchiopexy technique in rats leads to expression of Nitric oxide and stimulation of apoptosis in germ cells, which can be in concordance with the results of our study indicating infarction and necrosis after the occlusion of vessels^21^.

A study on histological and functional changes of the testis after ligature of the arteries, performed on adult and pre-pubertal rat models indicated testicular atrophy in 55% of young rats and inflammatory lesions in adult rats 85% of adult rats. Results of this study state that artery ligation can be responsible for the histological changes influencing the fertility of young and adult rats which is also in agreement with our results^22^.

Another research agreeing with our results is the study of the structure and the function of testis in rats that simultaneously underwent Fowler–Stephens orchiopexy with and opposite testicular orchiectomy. In this study, the level of serum testosterone, concentration of LDH and SDH enzymes, and testicular germ cells protein markers were measured at the time of killing the rats and in weeks 1, 2, 4, 6, 8, and 10. The results demonstrated that although the concentration of LDH and SDH in rats undergoing orchiopexy were decreased by 45% after 4 weeks; but in the control group, it was associated with a 5% increase. Serum testosterone level was reduced to a quarter of the basal level in the orchiopexy group, while it was slightly increased in the opposite group. Only a limited number of testes remained with good collateral blood supply and appropriate histological appearance^23^.

This current study did not evaluate the fertility state of the rats after the Fowler–Stephens technique which can be considered as a limitation for our results.

Kelly and colleagues studied the effect of Fowler–Stephens orchiopexy surgery technique on fertility in rats, using three groups of control, one-sided orchiopexy, and two-sided orchiopecy. They concluded that in the studied groups, fertility was not seen in the bilateral orchiopexy group. In the control group, fertility was complete and in the one-sided orchiopexy group fertility was present in 86% of cases^24^.

However, the main goal of the Fowler–Stephens orchiopexy is not to maintain fertility except in patients with bilateral intra-abdominal testes.

Considering that the Fowler–Stephens two-step orchiopexy technique is one of the ways to treat the undescended testis especially in cases with short testicular vessels or when the testis is immobile, the use of this technique becomes more popular. The problem with this technique is the probability of testicular ischaemia following the sudden closure of the testicular artery; although the collateral vessels prevent testicular atrophy but the tissue loses its germ cells during this stress and my no longer be functional.

The results of this study which aimed at modelling the Fowler–Stephens two-stage orchiopexy surgery technique in the rat and evaluated its effect on the histology of the testis, can be of use in terms of providing more knowledge about the advantages, disadvantages, and cost effectiveness of this method.

Although the results of this study have taken a step towards investigating the use of Fowler–Stephens surgical technique, but due to the limited sample size as well as the differences in laboratory and human models, more studies need to be done for more thorough and precise evaluation of this technique. It is suggested to conduct cohort studies on patients who have undergone this surgery to evaluate fertility outcomes and other important factors and to compare them with other surgical techniques.

## Conclusion

The results of our study show that abrupt closure of both testicular artery and vein in rats is associated with necrosis and infarction, decreased spermatogenesis and more inflammation in histopathological evaluations. However, no significant differences were found in terms of macroscopic findings including volume, consistency, and viability. Our findings indicate that the Fowler–Stephens orchiopexy technique should be done more cautiously in clinical practice. However, further investigations are essential to confirm the safety and long-term suitability of this technique on the viability and fertility of the testes.

## Ethical approval

This study was approved by the Ethics Committee with number 971169 and code IR.MUMS.MEDICAL.REC.1399.333.

## Consent

NA.

## Source of funding

This research did not receive any specific grant from funding agencies in the public, commercial, or not-for-profit sectors.

## Author contribution

Study conception and design: R.S., M.H., M.P.M. Data acquisition: R.S., M.H., M.P.M., P.B.T., S.K.A. , Y.J.M., L.A. Analysis and data interpretation: R.S., M.H., M.P.M., S.K.A., Y.J.M., L.A. Drafting of the manuscript: P.B.T. Critical revision: R.S., M.H., M.P.M., P.B.T.

## Conflicts of interest disclosure

None.

## Research registration unique identifying number (UIN)

This study was approved by the Ethics Committee of Mashhad University of Medical Sciences with number 971169.

## Guarantor

Mahdi Parvizi Mashhadi.

## Provenance and peer review

Not commissioned, externally peer-reviewed.

## Acknowledgements

I thank my colleagues and co-authors for their expertise and assistance throughout all aspects of our study and for their help in writing the manuscript.
